# Organic–Inorganic Modification of Magnesium Borate Rod by Layered Double Hydroxide and 3-Aminopropyltriethoxysilane and Its Effect on the Properties of Epoxy Resin

**DOI:** 10.3390/polym14173661

**Published:** 2022-09-03

**Authors:** Sai Zou, Li Dang, Ping Li, Jiachen Zhu, Shengjie Lan, Donghai Zhu

**Affiliations:** State Key Laboratory of Plateau Ecology and Agriculture, School of Chemical Engineering, Qinghai University, Xining 810016, China

**Keywords:** epoxy resin, magnesium borate rod, layered double hydroxide, composites, flame retardant

## Abstract

To alleviate the safety hazards associated with the use of epoxy resin (EP), a multifunctional filler was designed. This study firstly combines the superior mechanical properties of magnesium borate rods (MBR) with the excellent smoke suppression and flame-retardant characteristics of layered double hydroxide (LDH). H_2_PO_4_^−^ intercalated LDH (LDHP) was coated on the MBR surface to obtain inorganic composite particles MBR@LDHP. Subsequently, MBR@LDHP was modified with 3-aminopropyltriethoxysilane (APES) to obtain organic-inorganic composite particles MBR@LDHP-APES. Eventually, the hybrid particles were added to EP to prepare the composite materials. Thereafter, the morphology, composition, and structure of MBR@LDHP-APES were characterized utilizing scanning electron microscopy (SEM), Fourier transform infrared (FTIR), and X-ray diffraction (XRD). The results indicated the successful preparation of MBR@LDHP-APES, after which we investigated the effects of MBR@LDHP-APES on the smoke suppression, flame retardancy, and mechanical characteristics of EP. As observed, the EP composites containing 7.5 wt% MBR@LDHP-APES exhibited superior smoke suppression and flame retardancy abilities. The limiting oxygen index reached 33.5%, which is 36.73% greater than pure EP, and the lowest values of total heat and smoke release were observed for the composite materials. In addition, the mechanical properties test revealed that MBR@LDHP-APES considerably enhanced the tensile strength as well as the flexural strength of the composites. Furthermore, mechanistic studies suggested that the barrier effect of MBR, endothermic decomposition of LDHP, and the synergistic effect of LDHP and APES contributed essentially to the smoke suppression and flame-retardant properties of the material. The findings of this research point to a potential method for enhancing the EP’s ability to suppress smoke and flames while enhancing its mechanical properties.

## 1. Introduction

Epoxy resin (EP) is a vital thermosetting substance with several applications in coatings [1,2], adhesives [3,4], electronic appliances [5,6], structural applications [7,8], etc. However, it is flammable, smoke-prone, and exhibits inferior mechanical properties that can result in certain safety hazards during its application, which are subject to definite restrictions [9,10,11,12,13]. Studies have shown that dispersing various fillers in epoxy resins can address the above problems, and the effects of various fillers on the flame retardancy, the physicochemical, and the mechanical properties of epoxy composites are determined by many factors: the chemical nature of fillers, the surface properties of fillers, the amount of fillers added, the shape and size of their particles, the effect of fillers on the structure formation processes and the structure of the epoxy composites [14,15,16,17], etc. Therefore, the preparation of fillers that can improve the smoke suppression, flame retardancy, and mechanical properties of EP composites is of imminent significance.

In general, the composition and structure of layered double hydroxides (LDH) are flexible and controllable, and they are widely used in adsorption [18,19,20,21], catalysis [22,23,24], drug delivery [25,26], and flame retardancy [27,28,29,30,31], among other uses. In the current context, LDH has the proper characteristics to suppress smoke and prevent the spread of flames, as it absorbs a considerable amount of heat, and generates water vapor and metal oxides during its decomposition [32,33]. The conversion of anions between the hydrotalcite layers has garnered appreciable scholarly attention due to their capacity for enhancing the flame-retardant and smoke-suppressive characteristics of the composites. For instance, Xu et al. [34,35] used Mo_7_O_24_^6−^ and H_2_PO_4_^−^ intercalated hydrotalcite and observed a reduction in the heat and smoke release rates of the obtained composite material, with an increased oxygen index and carbon residue rate. However, LDH contains plenty of hydroxyl groups that can easily agglomerate because of its polar effect. Consequently, it is not uniformly dispersed in the polymer matrix, which in turn influences the flame-retardant efficiency of the composites to a certain extent [36]. In contrast, the addition of a large amount of LDH reduces the mechanical properties of the composite material [28,37]. Therefore, it is crucial to consider the composites’ mechanical characteristics to enhance their flame retardancy and smoke-suppressing abilities.

Studies have demonstrated that the addition of magnesium borate rods (MBR) can significantly enhance the mechanical properties of composite materials [38,39]. Moreover, it can be added to magnesium-aluminum alloys, ceramics, and polymers to improve the mechanical characteristics of the composite materials, including their impact strength, elastic modulus, and tensile strength [40,41]. Luo et al. [42] added ester-modified magnesium borate whiskers to a polypropylene matrix for preparing composites. The impact and tensile strengths of the produced composites were greater than pure PP composites. Similarly, Chen et al. [43] prepared magnesium borate whiskers/magnesium-based composites following the vacuum pressure infiltration method, which substantially improved the elastic modulus and tensile strength of the composites compared to the base alloy. However, MBR facilely agglomerates within the matrix, which impacts the performance of the material, since it is well known in the literature that aggregation phenomena led to a worsening of the thermomechanical properties of the composite [44]. More importantly, the lack of active groups on its surface raises the difficulty of its modification. Therefore, in the present study, the co-precipitation method is used to coat the MBR surface with H_2_PO_4_^−^ intercalated LDH (LDHP), wherein the MBR surface layer is enriched with active hydroxyl groups that can be conveniently modified. Simultaneously, it can attain improved smoke suppression, flame retardancy, and mechanical characteristics. Nonetheless, the composite particles (MBR@LDHP) obtained after inorganic modification exhibit strong hydrophilicity and weak compatibility with the polymer matrix. 

A silane coupling agent is an organosilicon compound with a special structure, comprising both a hydrolyzable group and an active group, which can interact with the organic functional group as well as the inorganic powder. This is a widely used coupling agent that displays adequate compatibility with the polymer matrix. More specifically, 3-aminopropyltriethoxysilane (APES) is an aminosilane coupling agent that is often used as a surface modifier. In addition, Si and N elements in APES can act as flame-retardant elements. In particular, Wang et al. [45] utilized APES to modify cellulose microcrystals and studied its flame retardancy with the reinforcing properties in EPs. For the EP composite derived by adding a certain amount of silane coupling agent-modified cellulose crystallites and organic phosphates to the EP, the heat release rate was reduced to 286 kW/m^2^ and the mechanical properties were significantly improved. Therefore, in the current study, the inorganic composite particles (MBR@LDHP) were modified with APES, the –Si–OH hydrolyzed by APES, and the –OH on the surface of the inorganic composite particles (MBR@LDHP), which were condensed into bonds to obtain the organic-inorganic composite particles (MBR@LDHP-APES). This aspect further improved the compatibility of the composite particles with the matrix and synergistically rendered the silicon, magnesium, aluminum, phosphorus, and nitrogen elements with flame retardancy.

**Scheme 1:**
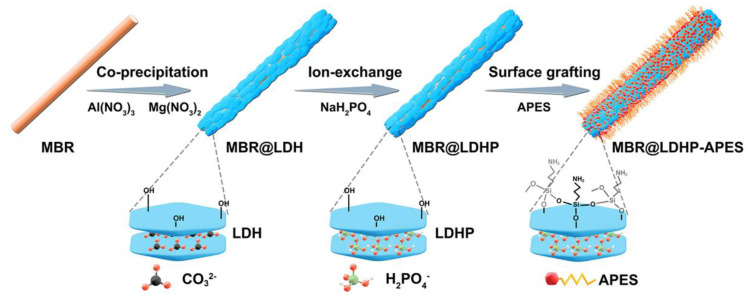
Representation of the synthesis process of MBR@LDHP-APES inorganic-organic hybrid.

In this study, we employed the co-precipitation method and ion exchange method to coat the surface of MBR with H_2_PO_4_^−^ intercalated magnesium aluminum hydrotalcite, which was subsequently modified with APES for addition to EP. The specific process is illustrated in Scheme 1, which aimed to combine the superior mechanical properties of the MBR with the excellent smoke-suppressive and flame-retardant characteristics of the LDHP, and the synergistic flame-retardant properties of silicon, phosphorus, magnesium, and other elements to yield EP composites with suitable mechanical, smoke suppression, and flame retardancy properties.

## 2. Experimental

### 2.1. Materials

The MBR was synthesized in our lab by combining the co-precipitation and sintering procedures, as was described in further detail in our earlier research [46]. Anhydrous sodium carbonate (Na_2_CO_3_, analytical reagent), aluminum nitrate nonahydrate (Al(NO_3_)_3_·9H_2_O, analytical reagent), sodium hydroxide (NaOH, analytical reagent), magnesium nitrate hexahydrate (Mg(NO_3_)_2_·6H_2_O, analytical reagent), sodium dihydrogen phosphate (NaH_2_PO_4_, analytical reagent), and nitric acid (HNO_3_, analytical reagent) were all purchased from Sinopharm Group Chemical Reagent Co., Ltd. (Shanghai, China). 3-aminopropyltriethoxysilane (APES, analytical reagent) and 4,4-diaminodiphenyl methane (DDM, analytical reagent) were acquired from Aladdin Biochemical Technology Co., Ltd. (Shanghai, China). Diglycidyl ether of bisphenol A (DGEBA, E44) was sourced from Nantong Xingchen Co., Ltd. (Jiangsu, China).

Table 1 shows the typical properties and specifications of DGEBA and DDM.

### 2.2. MBR@LDHP-APES Preparation

In a beaker, we put 10.0 g of MBR and 100.0 mL of deionized water, and then sonicated the mixture using a model KQ3200DB ultrasonication device (frequency: 40 kHz, power: 50 W, Kunshan Ultrasonic Instruments Co., Ltd., Kunshan, China) for 30 minutes. Thereafter, 1.2400 g of NaOH (Sinopharm Group Chemical Reagent Co., Ltd., Shanghai, China), 0.7949 g of Na_2_CO_3_ (Sinopharm Group Chemical Reagent Co., Ltd., Shanghai, China), and 150 mL of deionized water were used to produce solution A; 1.2821 g of Mg(NO_3_)_2_·6H_2_O (Sinopharm Group Chemical Reagent Co., Ltd., Shanghai, China), 1.8757 g of Al(NO_3_)_3_·9H_2_O (Sinopharm Group Chemical Reagent Co., Ltd., Shanghai, China), and 150 mL of deionized water were used to produce solution B. The three solutions were preheated to 70 °C, and the MBR slurry was stirred while adding the solutions A and B dropwise, controlling the pH to ~10, reacting at 70 °C for 30 min, and placing it in an oil bath at 70 °C overnight. Subsequently, the above-mentioned solution was sonicated under the same ultrasonic conditions for 15 minutes, NaH_2_PO_4_ solution was added dropwise, reacted at 60 °C for 2 h, filtered, rinsed with absolute ethanol (Sinopharm Group Chemical Reagent Co., Ltd., Shanghai, China), and water was added to produce a solution. An adequate amount of absolute ethanol, deionized water, and APES was pre-hydrolyzed for 1.5 h, gradually added dropwise to the above solution, stirred at 60 °C for 1 h, filtered with suction, and ultimately freeze-dried to obtain MBR@LDHP-APES. Furthermore, MBR@LDHP was prepared following the same method.

### 2.3. Preparation of EP Composites

The EP composite material was prepared following the physical mixing method. Specifically, in a 90 °C oil bath, the MBR@LDHP-APES was introduced into the DGEBA and vigorously stirred for 2 h. Thereafter, a predetermined amount of a curing agent (Aladdin Biochemical Technology Co., Ltd., Shanghai, China) was introduced. In addition, the bubbles were removed after 10 min, introduced into the mold, cured for 2 h at 100 °C, and aged for 2 h at 150 °C to produce the EP composites. In addition, the same method was used for preparing the EP composite materials added with MBR and MBR@LDHP (Table 2 presents the specific formulation).

### 2.4. Characterization

On a JSM 7900F field emission scanning electron microscope (FESEM; JEOL, Japan), images of the particles MBR, LDHP, MBR@LDHP, and MBR@LDHP-APES were captured using scanning electron microscopy (SEM). All samples were sprayed with gold for 15 s. The accelerating voltage was 10.0 kV at a working distance of 9.5 mm. Additionally, patterns of X-ray diffraction (XRD) were captured with diffraction peaks in the range of 5° to 90° on a D-max2500PC X-ray diffractometer (Rigaku Corp., Tokyo, Japan) equipped with Cu–K*α* radiation (λ = 0.154 nm). On a Nicolet 6700 spectrometer (Thermo Nicolet Corp., Madison, WI, USA), a Fourier-transform infrared (FTIR) spectroscopy was carried out to capture the distinctive peaks of MBR, LDHP, and APES. To measure the flame retardancy of pure EP and the composites based on ASTM D2863, a limiting oxygen index (LOI) measurement was performed utilizing a JF-6 oxygen index meter (Nanjing Xingguang Instrument Equipment Co., Ltd., Nanjing, China). In compliance with ISO 5660 and employing a heat flux of 35 kW/m^2^, the cone calorimeter test (CCT) was utilized to examine the flame- and smoke-suppressing capabilities of the EP composites and pure EP. The tensile properties of pure EP and composite materials were tested according to ASTM D638 (sample dimensions: 165 × 13 × 3 mm^3^) on an HD 021NS-5 tensile tester platform (Nantong Hongda Experimental Instrument Co., Ltd., Nantong, China) at a tensile speed of 1 mm min^−1^. The flexural properties were also tested on an HD 021NS-5 tensile tester platform according to GB/T 9341 (sample dimensions: 80×10×4 mm^3^). The mechanical properties of all the samples were measured five times, and the average values were reported and used in further studies. At a heating rate of 10 °C min^−1^, a DSC (Differential Scanning Calorimetry) 4000 differential scanning calorimeter (PerkinElmer Ltd., Shelton, CT, USA) was employed to evaluate the glass transition temperature (*T*_g_) of pure EP and the composites. Thereafter, a Netzsch STA449F3 analyzer (Netzsch Instrument Crop., Selbu, Germany) was utilized to carry out a thermogravimetric analysis (TG) at a heating rate of 10 °C/min from 25 to 850 °C under N_2_ atmosphere. Specifically, TG-FTIR uses a Perkin-Elmer STA6000 thermal analyzer (Perkin-Elmer Crop., Shelton, CT, USA) under a nitrogen atmosphere of 50 mL min^−1^, with the temperature rising from 40 to 800 °C at a heating rate of 10 °C min^−1^; the produced volatiles were flown into the Perkin–Elmer Frontier infrared spectrometer; a temperature of 260 °C was measured within the infrared cell; the resolution was 4 cm^−1^, the number of scans was 4, and the range of scanning was from 450 to 4000 cm^−1^. Furthermore, the primary source of illumination (λ = 532 nm) was an argon-ion laser, and on an RM2000 Raman spectrometer (Renishaw, UK), the Raman spectrum readings of the residual carbon were obtained. 

## 3. Results and Discussion

### 3.1. Characterization 

**Figure 1:**
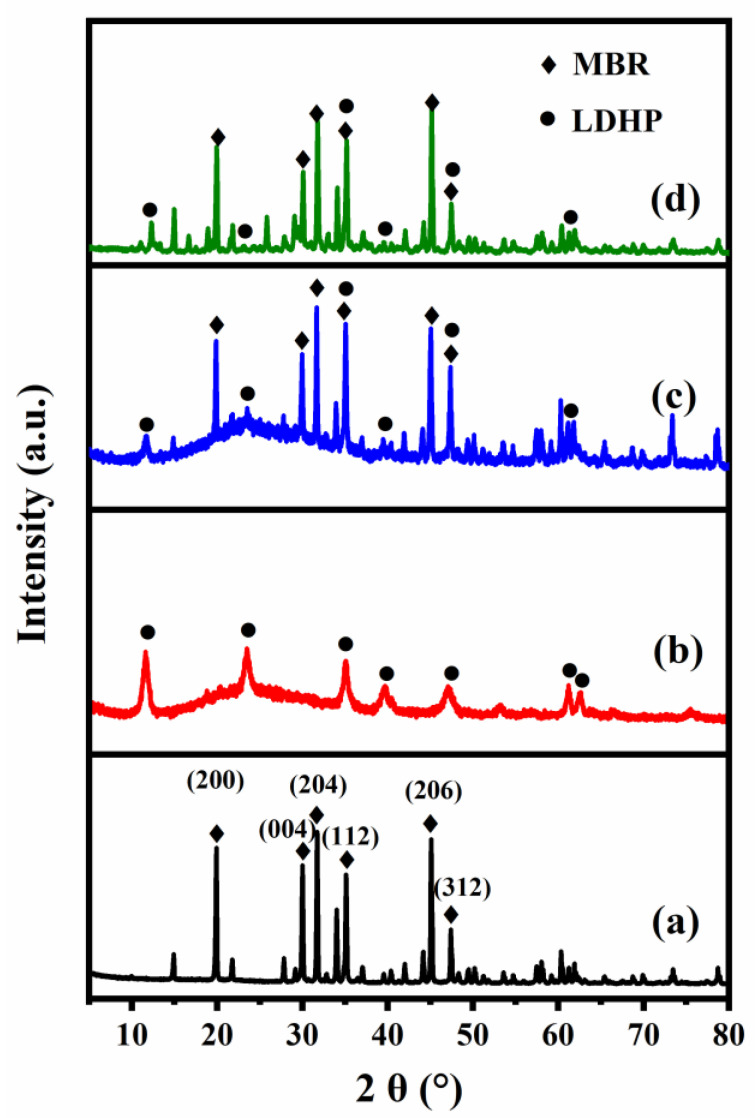
The XRD patterns for MBR (**a**), LDHP (**b**), MBR@LDHP (**c**), and MBR@LDHP-APES (**d**).

XRD measurements were used to describe the structures of the materials in their as-prepared states, and the XRD patterns of (a) MBR, (b) LDHP, (c) MBR@LDHP, and (d) MBR@LDHP-APES are illustrated in Figure 1. The primary characteristic diffraction peaks of MBR@LDHP at 19.91°, 29.95°, 31.71°, 35.06°, 45.11°, and 47.35° correspond to the (200), (004), (204), (112), (206), and (312) crystal plane of MBR, respectively [46]. The diffraction intensity of MBR@LDHP was inferior to that of MBR, which implies that the MBR surface was covered with substances. Moreover, the crystal planes of LDHP were observable in the XRD spectrum of MBR@LDHP [35], indicating that the LDHP was successfully coated on the MBR surface. The characteristic peaks of MBR@LDHP-APES were similar to MBR@LDHP, because the modification of MBR@LDHP by APES occurred only on the surface without appropriate crystallization of the APES, such that it did not modify the original crystal structure.

**Figure 2:**
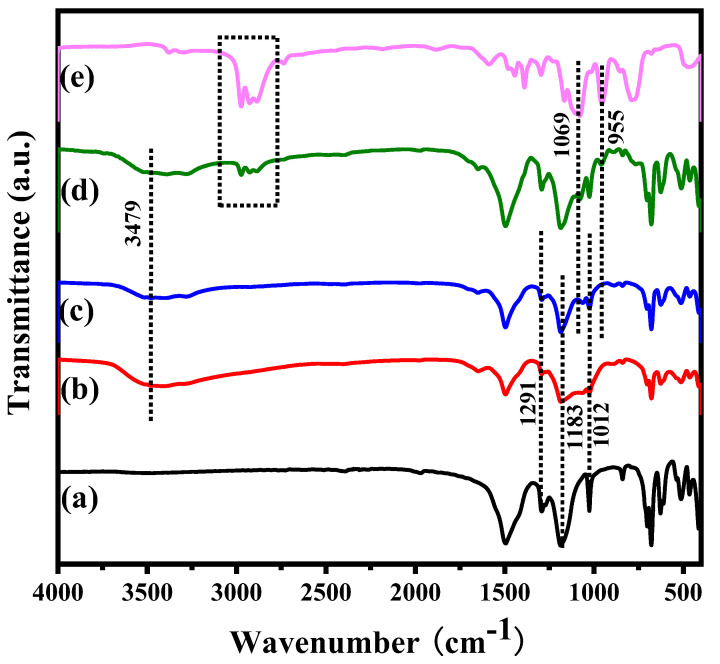
FTIR spectra of MBR (**a**), LDHP (**b**), MBR@LDHP (**c**), MBR@LDHP-APES (**d**), and DDP (**e**).

The structures of the as-prepared samples were characterized under infrared spectroscopy. The infrared spectra of (a) MBR, (b) LDHP, (c) MBR@LDHP, (d) MBR@LDHP-APES, and (e) APES are displayed in Figure 2. As portrayed in Figure 2a, the B–O and Mg–O stretching vibration bands [47], including the MBR surface, display almost no O–H stretching vibration peaks, thereby increasing the difficulty of direct organic modification. As depicted in Figure 2b, P=O and P–O stretching vibration peaks were detected at 1291, 1183, and 1012 cm^−1^, respectively, with no observable CO_3_^2−^ characteristic peak, indicating that the H_2_PO_4_^−^ successfully replaced CO_3_^2−^ intercalation LDH. As portrayed in Figure 2c, the characteristic peaks of MBR and LDHP were included as well, and the O–H peak of the stretching vibration was detected at 3479 cm^−1^. Thus, the LDHP was successfully coated on the MBR surface. According to the FTIR curve of APES in Figure 2e, the characteristic peak of N–H appeared at 1588 cm^−1^, the characteristic peak of Si–O–CH_2_ appeared at 1069 and 955 cm^−1^, and the C–H symmetrical stretching vibrations appeared in the vicinity of 2900 cm^−1^. As indicated in Figure 2d, the original characteristic peaks of MBR@LDHP and the C–H stretching vibration appeared near 2900 cm^−1^, and the characteristic peaks of Si–O–CH_2_ were detected at 1069 and 955 cm^−1^. However, the N–H functional groups could not be prominently identified because of the presence of the high-strength B-containing functional groups.

**Figure 3:**
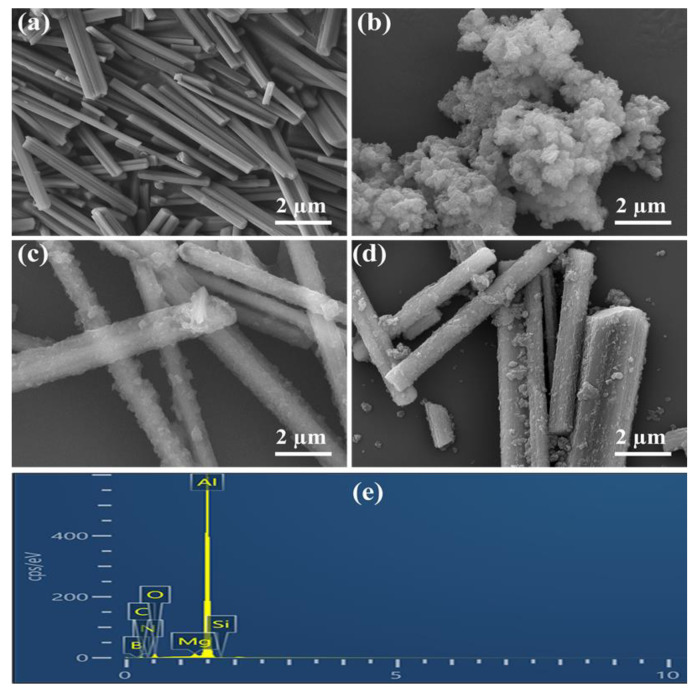
MBR images from SEM (**a**), LDHP (**b**), MBR@LDHP (**c**), and MBR@LDHP-APES (**d**); EDS spectrum of MBR@LDHP-APES (**e**).

The SEM images displaying the morphologies and structures of (a) MBR, (b) LDHP, (c) MBR@LDHP, and (d) MBR@LDHP-APES are depicted in Figure 3. As observed, MBR is a rod-like structure with a clean and smooth surface, whereas LDHP portrays a flower-like appearance, potentially because of the hydrogen bonds or other strong polar forces that accumulate. Compared to the MBR in Figure 3a, the MBR surface (Figure 3c) was wrapped with a continuous layer of material, implying the successful encapsulation of LDHP. The surface of the MBR@LDHP-APES (Figure 3d) appeared identical to the surface of the MBR@LDHP (Figure 3c), i.e., a rough surface. Nonetheless, the presence of Si and N elements in the EDS diagram, along with the findings presented in Figure 2 confirmed the successful preparation of MBR@LDHP-APES.

### 3.2. Thermal Stability of EP Composites

**Figure 4:**
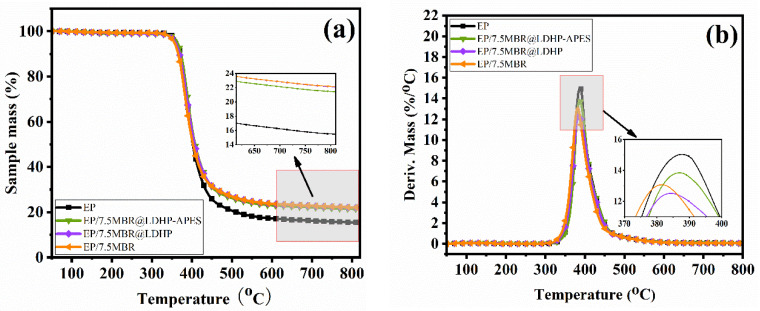
TG (**a**) and DTG (derivative thermogravimetry) (**b**) curves for EP and EP/7.5MBR@LDHP-APES, EP/7.5MBR@LDHP, EP/7.5MBR.

As illustrated in Figure 4, the TG tests on EP and its composites were carried out in an environment containing nitrogen, and the specific data are highlighted in Table 3. The TG curves of EP and its composites exhibited a similar trend, both involving single-step thermal degradation [48]. As evaluated, the *T*_−5%_ and *T*_max_ of EP were 363.7 °C and 386.5 °C, respectively. With the addition of various fillers, the *T*_−5%_ and *T*_max_ of EP composites reduced substantially in comparison with EP. Interestingly, the *T*_−5%_ and *T*_max_ of EP/7.5MBR@LDHP-APES were higher than those of EP/7.5MBR@LDHP and EP/7.5MBR, because the organically modified composite particles were more uniformly dispersed in the matrix, which has the potential to prevent the matrix from decomposing to a higher degree. However, the carbon residue of EP/7.5MBR was the largest, owing to the reasonable stability of the MBR hindering its ready decomposition. Compared to the carbon residue rates of EP, that of all composites were greater than EP, and that of EP/7.5MBR@LDHP-APES increased by 38.2%, which suggested the positive influence of APES and LDHP on promoting carbon formation.

**Figure 5:**
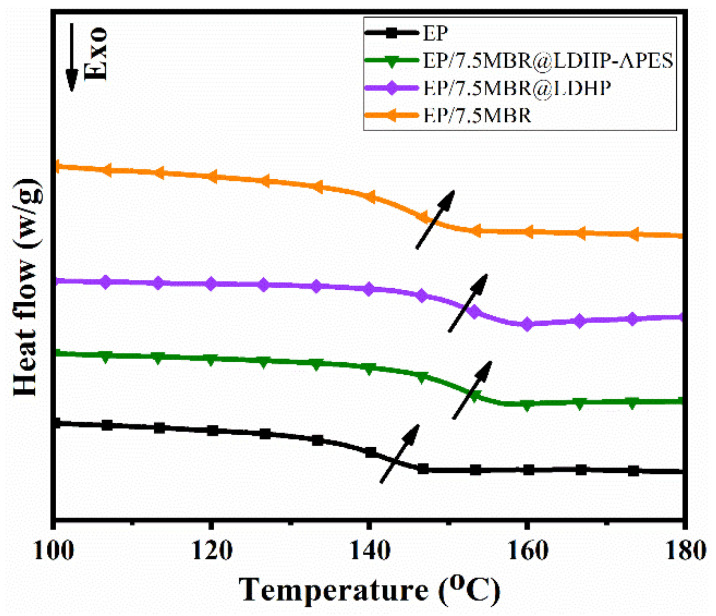
DSC curves for EP and EP/7.5MBR@LDHP-APES, EP/7.5MBR@LDHP, EP/7.5MBR.

Thereafter, we used DSC to examine the influence of various fillers on the *T*_g_ of EP under a nitrogen atmosphere of 30 mL min^−1^, in a temperature range from 20 to 200 °C at a heating rate of 10 °C min^−1^. Figure 5 displays a plot of the DSC curves for EP as well as the EP composites, wherein the *T*_g_ of EP was 143.3 °C. The *T*_g_ of the EP composites was shown to progressively rise following the introduction of several fillers. This may be because the hard fillers that were added to the composites acted to restrict the mobility of the EP chains. The *T*_g_ of EP/7.5MBR@LDHP-APES achieved a maximum temperature of 152.8 °C, which is 9.5 °C higher than that of EP.

### 3.3. EP Composite’s Mechanical Characteristics

**Figure 6:**
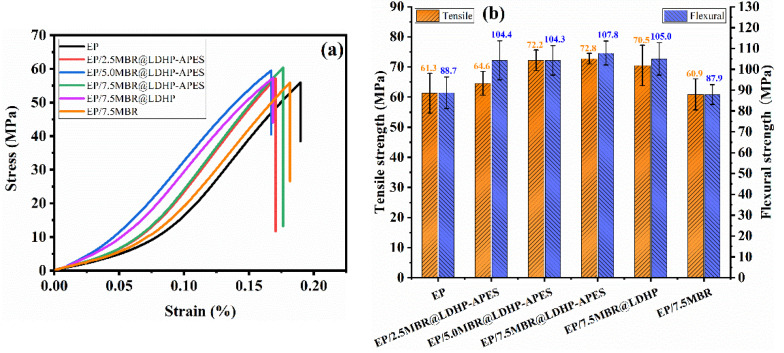
Tensile stress-strain curves (**a**) and tensile and flexural strength (**b**) of pure EP and its composites.

Because it is impossible to ignore the composite materials’ mechanical characteristics in real-world applications, this research examines the tensile and flexural strengths of the composite materials, as illustrated in Figure 6. Figure 6a shows the tensile stress-strain curves of pure EP and its composites; we found that the tensile strength and elastic modulus of the composites that had the modified MBR show greater improvement compared to that of pure EP. The tensile strength of pure EP was only 61.3 MPa and the flexural strength was 88.7 MPa. Accordingly, the tensile and flexural strengths of EP/2.5MBR@LDHP-APES, EP/5.0MBR@LDHP-APES, and EP/7.5MBR@LDHP-APES increased with the MBR@LDHP-APES content in the composite material. As the organic–inorganic composite particles were suitably compatible with the matrix, the composite particles were distributed evenly throughout the matrix, which also fulfilled the connection. Notably, upon adding 7.5wt%MBR@LDHP-APES to the matrix, the tensile and flexural strengths of EP/7.5MBR@LDHP-APES increased by 18.76% and 21.53% respectively, in contrast with the strength of pure EP, which accounts for the highest mechanical strength among the composites. However, the flexural and tensile strengths of EP/7.5MBR decreased by 0.9% and 0.65% compared to pure EP, respectively, because MBR exhibited inferior compatibility with the polymer matrix, and did not play a reinforcing role. In contrast with pure EP, the flexural and tensile strengths of EP/7.5MBR@LDHP displayed a substantial improvement, increasing by 18.38% and 13.05% respectively. However, they were not optimal, because the single inorganic modified composite particles exhibited weak compatibility with the matrix.

### 3.4. Flammability of EP Composites

**Figure 7:**
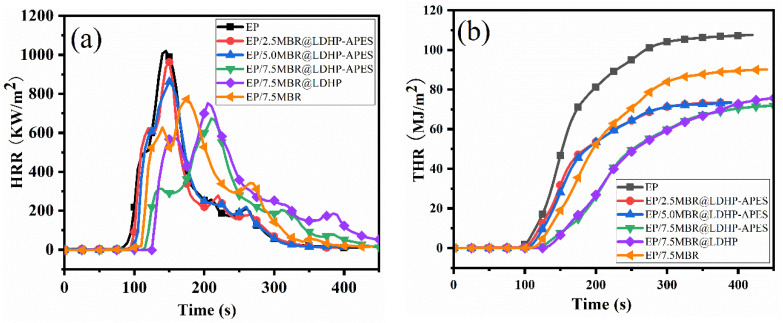
HRR (**a**) and THR(**b**) curves of pure EP and its composites.

The combustion performance of the composites was evaluated by examining their total heat release (THR) and heat release rate (HRR), as depicted in Figure 7, and the values are listed in Table 4. The THR and HRR of EP were 107.6 MJ·m^−2^ and 1020.2 kW·m^−2^, respectively, and the residual carbon rate was 10.3%; thus, the flame retardancy of EP is not particularly ideal. As the added amount of MBR@LDHP-APES increased, both the THR and HRR of the composites gradually decreased. The THR and HRR values of EP/7.5MBR@LDHP-APES were the lowest among the composites, i.e., 71.8 MJ·m^−2^ and 674.1 kW·m^−2^, respectively, which were 33.27% and 33.92% less than EP, respectively. Moreover, the carbon residue rate attained a maximum of value of 18.9%, which is 83.50% higher than that of EP, because the MBR forms a barrier on the surface of the composite material during combustion. Subsequently, the LDHP decomposes to produce oxides and water in an endothermic manner, and the intercalated H_2_PO_4_^−^ can generate PO· radicals, capturing hydrogen and hydroxyl radicals, thereby improving the flame retardancy of the composite material [49]. Compared with EP/7.5MBR@LDHP-APES, the HRR and THR values of EP/7.5MBR@LDHP and EP/7.5MBR increased to a certain extent owing to the lack of the synergistic flame retardant of APES, and the barrier impact of the generated SiO_2_ [50,51]. However, the heat release situation was better than pure EP; the HRR and THR of EP/7.5MBR@LDHP and EP/7.5MBR decreased by 26.27%, 24.28%, and 29.55%, 16.17% in comparison to those of EP, respectively. 

**Figure 8:**
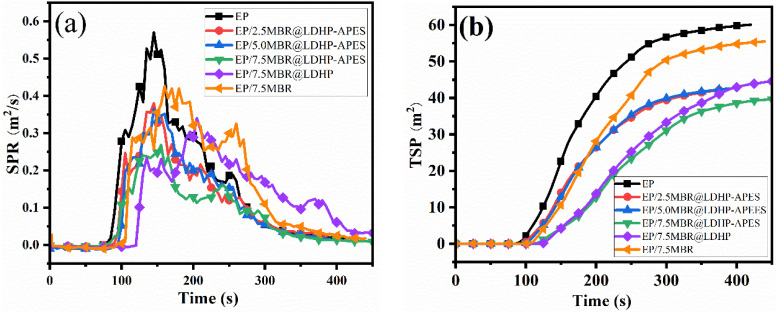
SPR (**a**) and TSP(**b**) curves of pure EP and its composites.

In addition, Figure 8 displays the smoke release rate (SPR) and total smoke release (TSP) of the composites. The SPR value and TSP value of EP were 0.57 m^2^·s^−1^ and 60.1 m^2^, respectively. As the added amount of MBR@LDHP-APES increased, the SPR and TSP values of the composite material progressively decreased. Overall, in comparison to the values of EP, the SPR and TSP values of EP/7.5MBR@LDHP-APES were the lowest, having decreased by 52.63 and 32.95%, respectively, due to the synergistic smoke suppression and flame retardancy of MBR, LDHP, and APES. However, the SPR and TSP values of EP/7.5MBR@LDHP and EP/7.5MBR exhibited an increase in comparison with EP/7.5MBR@LDHP-APES, respectively.

**Figure 9:**
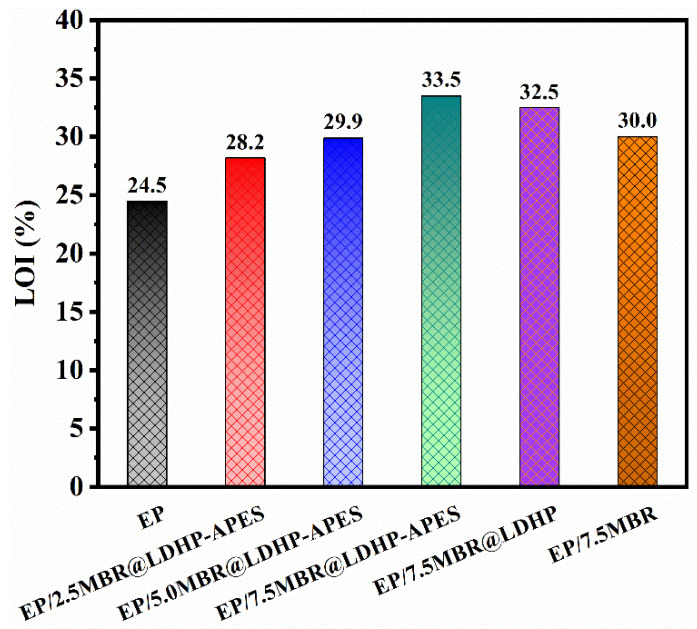
LOI of pure EP and its composites.

Figure 9 depicts the LOI values used to assess the composites’ flame retardancy. The LOI value of EP is 24.5%, and the flame-retardant effect was average. As the MBR@LDHP-APES content increased, the LOI of composites EP/2.5MBR@LDHP-APES, EP/5.0MBR@LDHP-APES, and EP/7.5MBR@LDHP-APES gradually increased. The LOI value of EP/7.5MBR@LDHP-APES was the highest (33.5%) because of the synergistic and efficient flame retardancy of magnesium, aluminum, silicon, and other flame-retardant elements, including the HO· and H· radicals freshly generated by combusting EP, which were trapped by the generated PO· radical [35,49,52]. The LOI values of EP/7.5MBR@LDHP and EP/7.5MBR were 32.5% and 30.0%, respectively, which were less than the LOI of EP/7.5MBR@LDHP-APES. These results indicate that the EP/7.5MBR@LDHP-APES composites exhibited superior fire safety and mechanical properties as compared to most of the composites reported previously (Table 5).

### 3.5. Mechanisms Linked to Flame Retardancy

**Figure 10:**
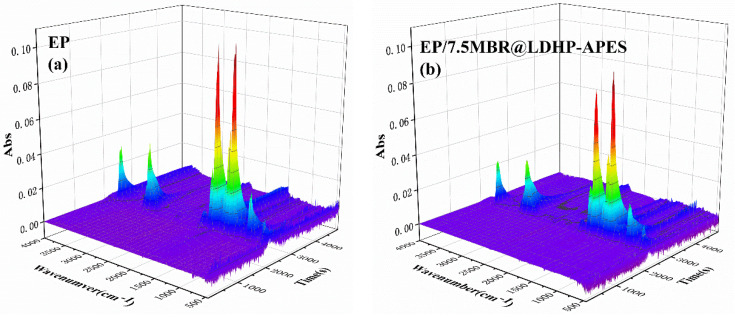
3D TG-FTIR of samples (**a**) EP and (**b**) EP/7.5MBR@LDHP-APES.

**Figure 11:**
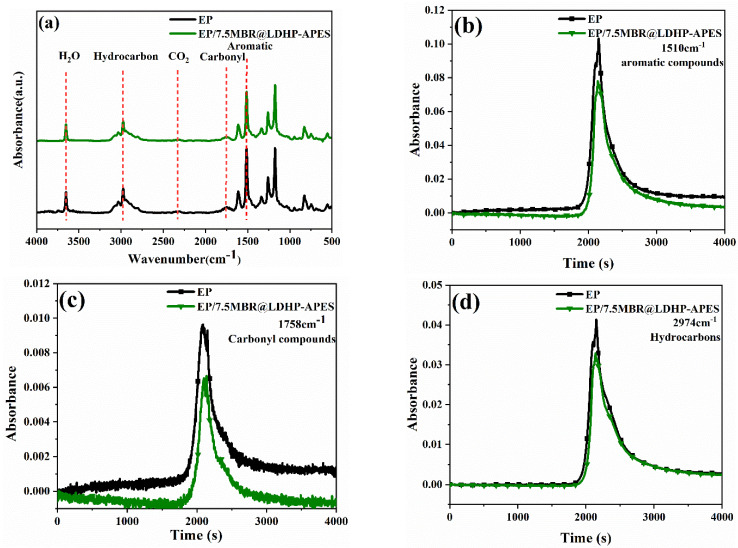
FTIR spectra of EP and EP/7.5MBR@LDHP-APES at the maximal rate of degradation (**a**); The FTIR absorption peak intensity pyrolysis products curve at: (**b**) 1510 cm^−1^, (**c**) 1758 cm^−1,^ and (**d**) 2974 cm^−1^ of EP and EP/7.5 MBR@LDHP-APES over time.

To further study the mechanisms behind the EP composites’ flame retardancy, the effect of MBR@LDHP-APES on the pyrolysis of EP was investigated through the TG-FTIR test. The 3D TG-FTIR spectra of EP and EP/7.5MBR@LDHP-APES cleavage products are illustrated in Figure 10a,b, respectively. The FTIR spectra of EP and EP/7.5MBR@LDHP-APES pyrolysis products at the maximum rate of evolution are illustrated in Figure 11a. As the infrared spectra of EP and EP/7.5MBR@LDHP-APES composites were almost identical, the pyrolysis components can be comparatively analyzed. The characteristic peaks of the aromatic compounds, carbonyl compounds, CO_2_, hydrocarbon groups, and H_2_O appeared at 1510, 1758, 2327, 2974, and 3648 cm^−1^ for EP and EP/7.5MBR@LDHP-APES, respectively [55]. Furthermore, the relationship between the FTIR absorption peak intensity and the time of the specific gas cracking products of EP and EP/7.5MBR@LDHP-APES composites is plotted in Figure 11b–d, respectively. As observed, the infrared absorption intensity of the pyrolysis products of EP/7.5MBR@LDHP-APES was reduced in contrast with that of EP. Therefore, adding fillers contributed substantially towards flame retardancy, and simultaneously, the dense carbon layer served as a stronger barrier, thereby reducing the release of the combustible organic compounds and delaying the evasion of the pyrolysis products. 

**Figure 12:**
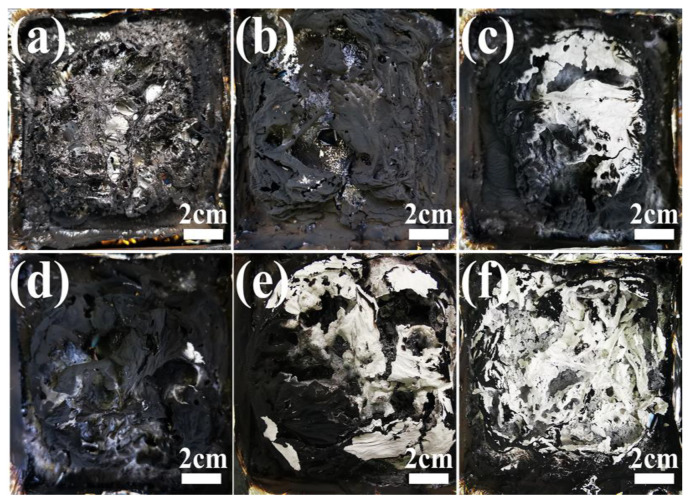
Digital images of EP char residue (**a**), EP/2.5MBR@LDHP-APES (**b**), EP/5.0 MBR@LDHP-APES (**c**), EP/7.5MBR@LDHP-APES (**d**), EP/7.5MBR@LDHP (**e**) and EP/7.5MBR (**f**).

The CCT carbon residue maps of pure EP and the composite materials are shown in Figure 12, which indicates the loose and porous carbon residue of the pure EP. Moreover, a marginal amount of carbon residue remained with only a thin layer. As the amount of MBR@LDHP-APES added increased, the residual carbon of EP/2.5MBR@LDHP-APES, EP/5.0MBR@LDHP-APES, and EP/7.5MBR@LDHP-APES gradually became more compact, and the number of voids diminished. This carbon layer can effectively prevent the entry of heat, and can concurrently isolate various flammable gases due to the enhanced synergistic effect of MBR, LDHP, and APES that ultimately promotes the formation of the carbon layer [31,35,56]. The carbon residues of EP/7.5MBR@LDHP and EP/7.5MBR were slightly improved in comparison to the residues of pure EP, owing to the addition of MBR@LDHP and MBR, respectively; however, they displayed inadequate compactness and stability. Among all the samples, the most prominent carbon residue was observed in the case of EP/7.5MBR, which was almost discontinuous, with a considerable amount of white matter floating on the surface. This confirmed the significance of inorganic coating and organic modification for MBR.

**Figure 13:**
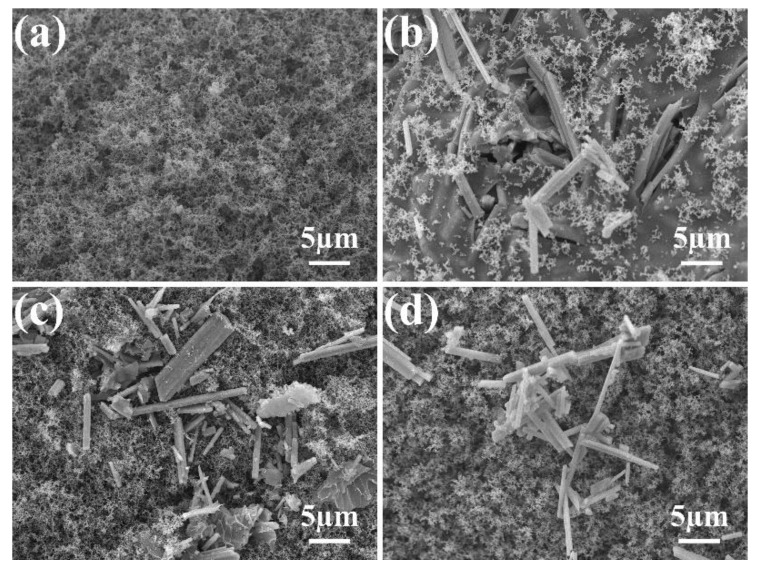
SEM images of char residues for EP (**a**), EP/7.5MBR@LDHP-APES (**b**), EP/7.5MBR@LDHP (**c**) and EP/7.5MBR (**d**).

The char residue microstructures for EP, EP/7.5MBR@LDHP-APES, EP/7.5MBR@LDHP, and EP/7.5MBR samples after the burning test were also observed by SEM (Figure 13). It was found that the char residue for the EP sample was very loose and had many holes. Conversely, the char residue for the EP/7.5MBR@LDHP-APES sample was denser and solider. The fillers were randomly distributed and bonded to the char residue to form a physical protective barrier. This continuous and intact char layer effectively blocks oxygen and external heat during combustion. For the EP/7.5MBR@LDHP sample, only a small amount of dense carbon was produced. From Figure 13d of the EP/7.5MBR sample, MBRs can be seen just physically piled together without adhesion. These loose and discontinuous char layers do not effectively prevent combustion from spreading into the interior of these samples.

**Figure 14:**
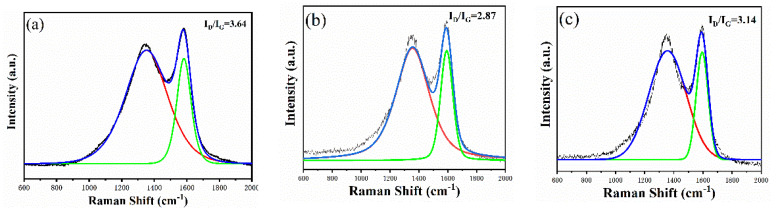
The char residue from EP examined using Raman spectroscopy. (**a**), EP/7.5MBR@LDHP-APES (**b**), and EP/7.5MBR@LDHP (**c**).

The Raman spectra of coke residues with EP, EP/7.5MBR@LDHP-APES, and EP/7.5MBR@LDHP are illustrated in Figure 14 for analyzing the structure and composition of cokes, including the mechanism of the action of flame retardants. In EP and its composites EP/7.5MBR@LDHP-APES and EP/7.5MBR@LDHP, graphitic D- and G-bands were detected at around 1350 and 1590 cm^−1^, respectively, corresponding to an amorphous carbon structure and an ordered graphite structure. The area ratio of the D-band to the G-band (I_D_/I_G_) may be used as a representation of the degree of graphitization that occurred in the residual carbon. Generally, a lower I_D_/I_G_ value represents a greater degree of graphitization, as well as a carbon layer that is highly agglomerated [57]. As observed, EP/7.5MBR@LDHP-APES reduced the I_D_/I_G_ from 3.64 to 2.87, whereas the I_D_/I_G_ value of EP/7.5MBR@LDHP was 3.14, which is only slightly decreased in comparison to EP. This was caused by the catalytic effect of Al_2_O_3_ and MgO generated during the decomposition of LDHP, which promoted carbon formation. After the introduction of APES, the formation of graphitic carbon as well as the compactness of the carbon layer further improved, thereby preventing the influx of heat as well as reducing the diffusion of combustible gases.

**Figure 15:**
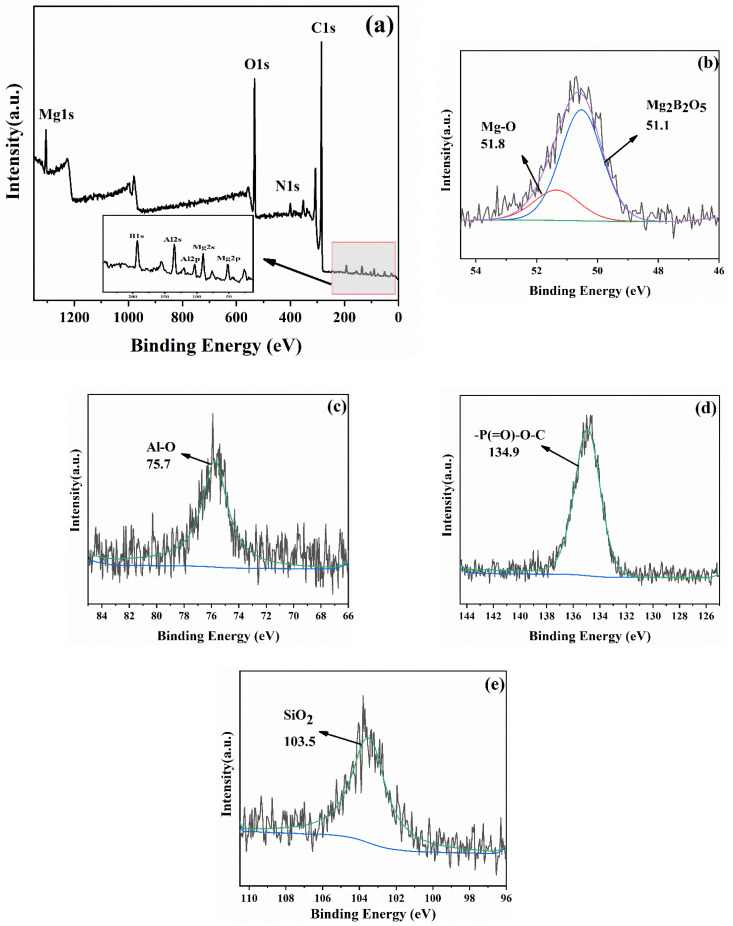
(**a**) Full-scan XPS spectral profiles of char residue derived from EP/7.5MBR@LDHP-APES, and high-resolution XPS spectral profiles obtained for (**b**) Mg2p, (**c**) Al2p, (**d**) P2p and (**e**) Si2p.

The char residue of EP/7.5MBR@LDHP-APES obtained after the CCT test was further investigated using the XPS technique. The full-scan XPS spectral profiles and high-resolution XPS maps of Mg2p, Al2p, P2p, and Si2p are presented in Figure 15. The signals of Mg2p, B1s, Al2p, P2p, N1s, and Si2p—absent in pure EP species—were detected in the char residue of EP/7.5MBR@LDHP-APES. As displayed in Figure 15b, the two peaks in the Mg2p spectrum at 51.1 and 51.8 eV corresponded to the interplay between Mg^2+^ and B_2_O_5_^4−^, and the Mg–O binding energy [53]. As depicted in the Al2p spectrum, the peak at 75.7 eV may be a result of the Al2p of Al–O (Al_2_O_3_) [54]. Thus, the Mg and Al elements in LDH generated MgO and Al_2_O_3_ during combustion, respectively. More importantly, the metal oxides form a physical barrier. In the P2p spectrum, a peak was detected at 134.9 eV, which may be a consequence of –P(=O)–O–C–. Thus, during combustion, H_2_PO_4_^−^ can generate a cross-linked structure of –P(=O)–O–C–, which consequently promotes carbonization and increases the density of the carbon layer. This results in an improvement in the composite’s smoke suppression and frame retardant abilities [35]. The Si2p spectrum exhibited a peak at 103.5 eV, which corresponded to SiO_2_ and indicated that the Si element in APES formed SiO_2_ during combustion. Thus, SiO_2_ formed a barrier to isolate the heat and combustible gases, which further delayed the decomposition of the matrix.

**Scheme 2:**
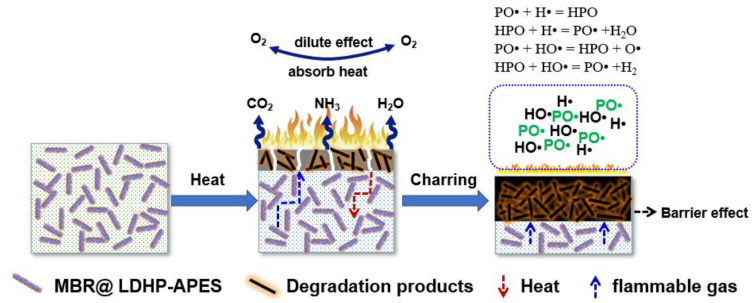
Depiction of the flame retardant and smoke suppression mechanisms exhibited by MBR@LDHP-APES in EP.

Following the research presented above, Scheme 2 provides a detailed summary of the flame retardant and smoke suppression mechanisms exhibited by MBR@LDHP-APES in EP. MBR@LDHP-APES enhanced the EP’s characteristics as a smoke suppressant as well as a flame retardant, primarily for the following reasons: the barrier effect of Mg_2_B_2_O_5_, MgO, Al_2_O_3_, and SiO_2_, the impact of water vapor and NH_3_ on dilution, the impact of the endothermic decomposition of LDH including catalytic carbon formation, and the PO· radicals generated by H_2_PO_4_^−^ which can capture H· or HO· during combustion. Furthermore, the N and Si elements in APES can synergize with Mg and Al elements for efficient flame retardation.

## 4. Conclusions

In conclusion, this research formulated organic–inorganic composite particles (MBR@LDHP-APES), and the characterization confirmed the uniform coating of the LDHP on the MBR surface and the successful grafting of the APES. Subsequently, MBR@LDHP-APES was added to EP to prepare the composites and study the properties of the materials. Compared to pure EP, EP/7.5MBR@LDHP-APES exhibited improved flame retardancy and smoke suppression characteristics, and the THR, PHRR, TSP, and PSPR of EP/7.5 MBR@LDHP were reduced by 33.27%, 33.92%, 32.95%, and 52.63%, respectively. The LOI and coke yields of EP/7.5MBR@LDHP-APES were 33.5% and 18.90%, which were 36.73% and 83.50% higher than that of EP, respectively. This may be caused by the barrier effect of MBR, the barrier and adsorption effects of MgO, SiO_2_, and Al_2_O_3_, the influence that water vapor has on diluting the mixture, as well as the endothermic influence of MH breakdown. Thus, the APES and inorganic flame retardants synergistically induced flame retardancy. Compared to EP, the mechanical properties of EP/7.5MBR@LDHP-APES were improved, as the tensile and flexural strengths were increased by 18.76% and 21.53%, respectively. Therefore, this research established a potential avenue for enhancing the flame retardancy, smoke repression, and mechanical properties of composite materials.

## Figures and Tables

**Table 1:** The typical properties and specifications of DGEBA and DDM.

The Qualitative Characteristics	Value
Properties of DGEBA	
Density at 25 °C, g/cm^3^	1.18
Viscosity at 25 °C, Pa s	20–40
softening point, °C	14–23
Epoxy equivalent, (g mol^−1^)	210–230
epoxy value, mol 100g^−1^	0.41–0.48
Properties of DDM	
Molecular mass, g mol^−1^	198.28
Density at 25 °C, g/cm^3^	1.15
Viscosity at 25 °C, Pa s	2.5–4
melting point/freezing point, °C	91.5–92
Amine value (mg KOH g^−1^)	480

**Table 2:** Pure EP preparations and their composites.

Sample	DGEBA (wt%)	DDM (wt%)	MBR (wt%)	MBR@LDHP (wt%)	MBR@LDHP-APES (wt%)
EP	82.0	18.0	0	0	0
EP/2.5 MBR@LDHP-APES	80.0	17.5	0	0	2.5
EP/5.0 MBR@LDHP-APES	78.0	17.0	0	0	5.0
EP/7.5 MBR@LDHP-APES	75.8	16.7	0	0	7.5
EP/7.5 MBR@LDHP	75.8	16.7	0	7.5	0
EP/7.5 MBR	75.8	16.7	7.5	0	0

**Table 3:** TG data for EP and its composites.

Sample	*T*_−5%_ (°C)	*T*_max_ (°C)	Char Yield (%)
EP	363.7	387.4	15.49
EP/7.5MBR@LDHP-APES	363.6	386.7	21.41
EP/7.5MBR@LDHP	359.1	383.9	22.10
EP/7.5MBR	356.1	380.8	22.15

**Table 4:** CCT test results for EP and its composites.

Sample	PHRR	THR	Mass	SPR	TSP
	(KW·m^−2^)	(MJ·m^−2^)	(%)	(m^2^·s^−1^)	(m^2^)
EP	1020.2	107.6	10.3	0.57	60.1
EP/2.5 MBR@LDHP-APES	962.6	73.4	15.9	0.38	42.6
EP/5.0 MBR@LDHP-APES	861.6	73.4	16.2	0.37	42.8
EP/7.5 MBR@LDHP-APES	674.1	71.8	18.9	0.27	40.3
EP/7.5MBR@LDHP	752.2	75.8	18.2	0.34	46.3
EP/7.5MBR	772.5	90.2	17.8	0.42	55.5

**Table 5:** Combustion and mechanical properties of previously reported EP composites.

Composite (Content wt%)	Flame Retardancy			Mechanical Properties		Ref.
	PHRR	PSPR	LOI	Tensile Strength	Flexural Strength	
EP/APP (11.0)	−42.8%	No given	17.9%	−43.5%	No given	[45]
EP/MH (7.5)	−16.3%	−30.6%	14.6%	−23.3%	No given	[53]
EP/LDH (2.0)	−21.3%	−12.9%	14.2%	−3.7%	−9.7%	[9]
EP/LDH (7.5)	−16.1%	−30.6%	16.7%	−20.2%	No given	[54]
EP/MBR@LDHP-APES (7.5)	−33.9%	−52.6%	36.7%	18.8%	21.5%	This work

## Data Availability

The data used to support the findings of this study are available from the corresponding author upon request.
